# The HIFα-Stabilizing Drug Roxadustat Increases the Number of Renal Epo-Producing Sca-1^+^ Cells

**DOI:** 10.3390/cells11040753

**Published:** 2022-02-21

**Authors:** Aline Jatho, Anke Zieseniss, Katja Brechtel-Curth, Jia Guo, Kai Oliver Böker, Gabriela Salinas, Roland H. Wenger, Dörthe M. Katschinski

**Affiliations:** 1 Institute of Cardiovascular Physiology, University Medical Center Göttingen, Georg-August-University Göttingen, 37073 Goettingen, Germany; anke.zieseniss@med.uni-goettingen.de (A.Z.); katja.brechtel-curth@med.uni-goettingen.de (K.B.-C.); jia.guo@med.uni-goettingen.de (J.G.); 2 Department of Trauma Surgery, Orthopaedics and Plastic Surgery, University Medical Center Göttingen, Georg-August-University Göttingen, 37075 Goettingen, Germany; kai.boeker@med.uni-goettingen.de; 3 NGS-Integrative Genomics Core Unit (NIG), Institute of Human Genetics, University Medical Center Göttingen, Georg-August-University Göttingen, 37073 Goettingen, Germany; gsalina@gwdg.de; 4 National Centre of Competence in Research “Kidney.CH”, 8057 Zurich, Switzerland; roland.wenger@access.uzh.ch; 5 Institute of Physiology, University of Zürich, 8057 Zurich, Switzerland

**Keywords:** HIFα-stabilizing drugs, PHD inhibitor, roxadustat, erythropoietin, Sca-1

## Abstract

Inhibition of the prolyl-4-hydroxylase domain (PHD) enzymes, leading to the stabilization of hypoxia-inducible factor (HIF) α as well as to the stimulation of erythropoietin (Epo) synthesis, is the functional mechanism of the new anti-anemia drug roxadustat. Little is known about the effects of roxadustat on the Epo-producing cell pool. To gain further insights into the function of PHD inhibitors, we characterized the abundance of mesenchymal stem cell (MSC)-like cells after roxadustat treatment of mice. The number of Sca-1^+^ mesenchymal cells following roxadustat treatment increased exclusively in the kidneys. Isolated Sca-1^+^ cells demonstrated typical features of MSC-like cells, including adherence to tissue culture plates, trilineage differentiation potential, and expression of MSC markers. Kidney-derived Sca-1^+^ MSC-like cells were cultured for up to 21 days. Within the first few days in culture, cells stabilized HIF-1α and HIF-2α and temporarily increased Epo production upon incubation in hypoxia. In summary, we have identified a Sca-1^+^ MSC-like cell population that is involved in renal Epo production and might contribute to the strong anti-anemic effect of the PHD inhibitor roxadustat.

## 1. Introduction

The oxygen-sensitive hypoxia-inducible factor (HIF) pathway plays a central role in cellular adaptation to limited oxygen supply [1]. The protein stability of the HIFα subunit is regulated by oxygen- and iron-dependent prolyl-4-hydroxylase domain (PHD) enzymes [2]. Local tissue hypoxia is a major factor triggering erythropoietin (Epo) production [3]. Epo inhibits apoptosis of erythroid progenitor cells, which results in an increase in red blood cells and improved oxygen transport capacity. The hypoxic induction of Epo is tightly connected to the HIF pathway, since Epo is almost exclusively regulated on the transcriptional level via the binding of HIF to various regulatory elements of the *Epo* gene [4]. The kidney is the main site of Epo synthesis in adults. Pathological conditions such as chronic kidney disease (CKD) impair the renal ability to synthesize Epo, resulting in hypoproliferative renal anemia. The administration of recombinant Epo represents the current standard of care for patients with renal anemia [5].

The discovery of druggable PHD enzymes has spurred the development of novel therapeutic agents for the treatment of renal anemia. PHD inhibitors (PHIs) promote coordinated erythropoiesis through increasing endogenous Epo expression by blocking the degradation of HIFα [6]. These compounds effectively stimulate erythropoiesis in CKD patients. Several clinically applicable PHIs have been developed. The approval decision for roxadustat (FG-4592) was first granted in China for CKD anemia in December 2018 [7] and subsequently in Japan. Molidustat (Bay-85-3934) was approved in Japan in 2021. Vadadustat (AKB-6548) and desidustat (ZYAN1) are currently undergoing clinical investigations. In terms of approaches to therapy, the specificity of conventional recombinant Epo is in striking contrast to the pleiotropic effects of activating the HIF pathway, considering that Epo is just one HIF target gene among hundreds. However, the broad hypoxia-mimicking effect of PHIs might also be useful for therapeutic indications other than the management of renal anemia, such as tissue protection in ischemic diseases [8].

The nature, origin, and especially plasticity of the renal Epo-producing (REP) cells under resting as well as stimulated conditions are still under debate. Under non-stimulated conditions, REP cells are located in the peritubular interstitial space of the corticomedullary border region [9]. In hypoxia, the majority of cortical perivascular fibroblasts/pericytes have the capacity to produce Epo. These cells can be found throughout the entire renal cortex and outer medulla, indicating that REP cells represent a diverse cell pool with high plasticity [10,11]. The difficulties of establishing a kidney-derived REP cell line have hampered the Epo field for quite some time. Altogether, emerging data indicate that at least some of the REP cells stem from mesenchymal-like progenitors with features shared by fibroblasts, pericytes, and telocytes [12]. It remains unknown, however, when and upon which stimulus these cells play a role in endogenous Epo production. In adults, mesenchymal cells are located in the interstitium or stroma of most tissues and retain at least part of their embryonic plasticity. The expression of the surface marker stem cell antigen-1 (Sca-1) is typical for mesenchymal cells in mice. The Sca-1 protein was first identified as a member of the Ly-6 gene superfamily in the 1970s, and is hence also known by its alternative name, Ly-6A/E [13]. Recently, conditionally immortalized cell lines derived from REP cells from Epo reporter mice were successfully established. Under non-proliferative and neurogenic conditions, these cells underwent a marked upregulation of HIF-2α but not HIF-1α mRNA, and acquired a stem-cell-like state, with strongly enhanced Sca-1 and CD133 (prominin-1) induction [14]. To gain further insight into the Epo-stimulating properties of the PHIs, we treated mice with roxadustat. We observed an increase in Sca-1^+^ cell numbers in the kidneys and identified an MSC-like cell population that has the ability to produce Epo.

## 2. Materials and Methods

### 2.1. Animals

C57BL/6 mice were treated by intraperitoneal (i.p.) injection with roxadustat (33 mg/kg body weight in 0.5 M Tris-HCl, pH 9; Selleckchem, Munich, Germany) for seven or 14 days. Control mice were treated with the solvent without adding roxadustat. All animal work conformed to institutional guidelines by the Niedersächsische Landesamt für Verbraucherschutz und Lebensmittelsicherheit (approval number: 33.9-42502-04-14/1498).

### 2.2. Isolation of Mesenchymal Cells and Magnetic Cell Separation (MACS)

Mouse kidneys were harvested, washed with PBS, and thoroughly minced. The homogenates were treated with 4% trypsin (P10-027100, PAN-Biotech, Aidenbach, Germany) and collagenase IV 1450 U/mL (Merck Millipore, Darmstadt, Germany) in Hank’s balanced salt solution for 1 h, filtered subsequently through 150, 70, and 40 µm cell strainers (Corning) and collected in PBS, 2% FCS (PAN Biotech), or PBS, 0.5% albumin faction V (Applichem, Darmstadt, Germany) for further magnetic cell separation (MACS) or FACS analysis. Isolated mesenchymal cells were MACS-sorted using a Sca-1 antibody (Biotin Ly-6a/e Clone D7, BD Pharmingen, Heiddelberg, Germany, dilution 1:200)/streptavidin magnetic bead (130-048-101, Miltenyi Biotec, Bergisch Gladbach, Germany) combination and XS columns (130-041-202, Miltenyi Biotech) according to the vendor’s instructions.

### 2.3. Cell Culture of Sca-1^+^ Cells

MACS-isolated Sca-1^+^ cells were cultured in Mesenpan cell culture medium (PAN-Biotech) complemented with 2% FCS and 1% antibiotics (PAN-Biotech). For hypoxic incubation (1% O_2_, 5% CO_2_), cells were incubated in an InvivO_2_ 400 workstation (Baker Ruskinn, Sanford, ME, USA).

### 2.4. Human Bone Marrow-Derived Mesenchymal Stem Cells

Human bone marrow-derived mesenchymal stem cells (MSC) were isolated from a proximal femur after total hip replacement surgery. All experiments were conducted according to ethical principles, including the World Medical Association Declaration of Helsinki. Isolation of MSC and further experiments were approved by the ethics committee of the Universitätsmedizin Göttingen, Georg-August-Universität Göttingen, Germany, reference number 10/12/17. Human bone marrow was mixed with 10 mL DMEM medium (Thermo Fisher, Waltham, MA, USA), filtered through a 100-nm cell strainer (Thermo Fisher) and digested by collagenase I (0.25%) for 1 h at 37 °C. Cells were centrifuged at 500× *g* for 5 min and pellet was transferred into six-well plates. Cells were cultivated at 37 °C and 5% CO_2_.

### 2.5. Tri-Lineage Differentiation

Sca-1^+^ cells were cultured and expanded for seven days. The cell culture medium was exchanged using the respective differentiation medium for osteogenesis (Promocell, Heidelberg, Germany C-28013), adipogenesis (Promocell, C-28016), or chondrogenesis (Promocell, C-28012) and maintained for 14, 21, and again 21 days, respectively.

### 2.6. Cell Culture of L929 Cells

L929 cells were obtained from the American Type Culture Collection, Manassas, VA, USA. Cells were cultivated in high-glucose modified Eagle’s medium (PAN-Biotech) supplemented with 10% fetal calf serum (Biochrom, Berlin, Germany), 50 units/mL penicillin, and 50 µg/mL streptomycin (PAN-Biotech). For obtaining a conditioned medium, L929 cells were cultivated in a Mesenpan cell culture medium (PAN-Biotech) supplemented with 2% FCS and 1% antibiotics (PAN-Biotech).

### 2.7. ELISA

Epo concentrations in cell culture supernatants and plasma samples were quantified using the mouse Epo Quantikine ELISA Kit (MEP00B, R&D Systems, Wiesbaden, Germany) according to the vendor’s instructions.

### 2.8. Protein Extraction and Western Blot Analysis

Cells were lysed in 10 mM Tris-HCl pH 8.0, 400 mM NaCl, 1 mM EDTA, 0.1% Triton-X100, supplemented with a protease inhibitor cocktail (cOmplete Mini, Roche Applied Science, Mannheim, Germany). For Western blot analysis, the following primary and secondary antibodies were used: anti-HIF-1α (NB100-479, Novus, Wiesbaden Germany; dilution 1:1000), anti-HIF-2α (AF2997, R&D Systems; dilution 1:2000), anti-β-tubulin (ab15246, Abcam, Cambridge, UK; dilution 1:1000), goat anti-rabbit IgG HRP (sc-2004, Santa Cruz Biotechnologies, Dallas, TX, USA; dilution 1:10,000), and mouse anti-goat IgG HRP (sc-2354, Santa Cruz Biotechnologies; dilution 1:2000).

### 2.9. FACS Analysis

Cells were incubated with APC/Cy7 anti-mouse Ly-6A/E (Sca-1) (BioLegend, San Diego, CA, USA, 108125, dilution 1:200), FITC anti-mouse CD117 (c-Kit) (BioLegend, 105805; dilution 1:200), AlexaFluor488 anti-mouse PDGFRβ (Becton Dickinson, Heidelberg, Germany 558427, dilution 1:200), and Hoechst 33342 (Sigma Aldrich, Hamburg, Germany; dilution 1:10,000) for 1 h at 4 °C in the dark. For the proliferation analysis, MACS-sorted cells were fixed with chilled ethanol and incubated at 20 °C for 2 h followed by APC anti-mouse Ki67 (BioLegend, 652405, dilution 1:200) and Hoechst for 30 min at room temperature (RT) in the dark. For the apoptosis analysis, MACS-sorted cells were resuspended in annexin V binding buffer (BioLegend, 422201) and stained with Annexin V 647 (BioLegend, 640911, dilution 1:20) and Zombie Green (BioLegend, 423111, dilution 1:1000) for 10 min in the dark. Samples were washed twice with PBS and analyzed by flow cytometry (BD FACS Canto II and BD FACS Aria III).

### 2.10. RNA Isolation and Quantitative RT-PCR

Total RNA was isolated using TRIzol (Thermo Fisher Scientific) and reverse-transcribed (RT) into cDNA using the Revert Aid First strand cDNA synthesis kit (Thermo Fisher Scientific). Quantitative PCR was performed using a SensiMix SYBR Low-ROX kit (Bioline, Luckenwalde, Germany) according to the manufacturer’s instructions. PCR products were quantified by comparison to a standard curve or analyzed by the ΔΔCT method compared to rpL28 as a reference gene.

### 2.11. Primer Sequences

Arginase for 5′-AGGACAGCCTCGAGGAGGGG-3′, rev 5′-CCCTGGCGTGGCCAGAGATG-3′; BNIP3 for 5′-GTCCAGTGTCGCCTGGCCTC-3′; rev 5′-TGGGAGCGAGGTGGGCTGTC-3′; CAIX for 5′-GGGGTCATCTGGACTGTGTT-3′, rev 5′-CCCACTTCTGTGCCTGTGCT-3′; Epo for 5′-AATGGAGGTGGAAGAACAGG-3′, rev 5′-ACCCGAAGCAGTGAAGTGA-3′; IL-6 for 5′-GCTGGTGACAACCACGGCCT -3′, rev 5′-TGCACAACTCTTTTCTCATTTCCACGA-3′; L28 for 5′-GCAAAGGGGTCGTGGTAGTT-3′, rev 5′-TTCTGGCTTCGAAGGATGGC-3′; PAI1 for 5′-CCAACATCTTGGATGCTGAA-3′, rev 5′-CTGCTCTTGGTCGGAAAGACT-3′; PHD2 for 5′-TTGCTGACATTGAACCCAAA-3′, rev 5′-GGCAACTGAGAGGCTGTAGG3′; PHD3 for 5′-GGCCGCTGTATCACCTGTAT-3′, rev 5′-TTCTGCCCTTTCTTCAGCAT-3′; TNFα for 5′-GACCCTCACACTCAGATCATCTTC-3′, rev 5′-CCACTTGGTGGTTTGCTACGA-3′; YM1 for 5′-GCCAGCAGAAGCTCTCCAGAAGCAA-3′, rev 5′-ACTGAACGGGGCAGGTCCAAACT-3′.

### 2.12. Transcriptome and Bioinformatic Analysis

#### 2.12.1. RNAseq Library Preparation

The quality and integrity of RNA were assessed with a fragment analyzer (Advanced Analytical, Heidelberg, Germany) using a standard sensitivity RNA analysis kit (DNF-471). All samples selected for sequencing exhibited an RNA integrity number over 8. RNAseq libraries were generated using 500 ng total RNA of a nonstranded RNA Seq, massively-parallel mRNA sequencing approach from Illumina (TruSeq stranded total RNA Library Preparation, Illumina, San Diego, CO, USA). Libraries were prepared on the automation workstation (Beckman Coulter’s Biomek FXP, Krefeld, Germany). For accurate quantitation of cDNA libraries, the fluorometric based QuantiFluor™ dsDNA system from Promega, Madison, WI, USA was used. The size of the final cDNA libraries was determined using the dsDNA 905 reagent kit (Fragment Analyzer), with a size of 300 bp on average. Libraries were pooled and sequenced on the Illumina HiSeq 4000 (SE; 1 × 50 bp; 30–35 Mio reads/sample). Sequence images were transformed with the Illumina software BaseCaller into BCL files, which were demultiplexed to fastq files with bcl2fastq v2.17.1.14. The quality check was done using FastQC version 0.11.5 (https://www.bioinformatics.babraham.ac.uk/projects/fastqc/, accessed on 17 February 2022).

#### 2.12.2. Mapping and Normalization

Sequences were aligned to the genome reference GRCm38 (mm10) sequence using the STAR aligner [15]. Subsequently, read counting was performed using featureCounts [16]. Read counts were analyzed in the R/Bioconductor environment (version 3.4.2) using the DESeq2 package version 1.14.1. Candidate genes were filtered using an absolute log2 fold-change > 1 and FDR-corrected *p*-value < 0.05. Gene annotation was performed using *Homo sapiens* entries via biomaRt R package version 2.32.1 [17].

### 2.13. Macrophage Isolation and Differentiation

Bone marrow-derived macrophages (BMDM) were isolated and differentiated as described previously [18]. Adherent BMDM were detached with 3.5 mL accutase (PAA Laboratories, Cölbe, Germany) and resuspended in culture medium (DMEM, supplemented with 0.2 mM L-glutamine, 0.1 mM sodium pyruvate, 1 mM HEPES, 50 U/mL penicillin G, 50 µg/mL streptomycin, and 10% heat-inactivated FCS). For M1- and M2-polarization, cells were stimulated with 100 ng/mL LPS (ALX-581-013-L001, Enzo Life Sciences, Lörrach, Germany) and 20 nM IFN-γ (315-05, Peprotech, Hamburg, Germany) or 20 nM IL-4 (214-14, Peprotech) for 24 h, respectively. Stimulation was either performed in a conditioned medium from L929 cells or a conditioned medium obtained from Sca-1^+^ cells.

### 2.14. Statistical Analysis

The statistical significance of the difference between two sample groups was calculated by an unpaired two-tailed Student’s *t*-test. In the case of more than two groups, all were tested by a one-way analysis of variance (ANOVA). Data are shown as the mean ± standard error of the mean (SEM). A significant difference between two means was defined as a *p*-value < 0.05.

## 3. Results

### 3.1. Roxadustat Treatment Increases the Number of Sca-1-Positive Cells in the Kidneys

**Figure 1:**
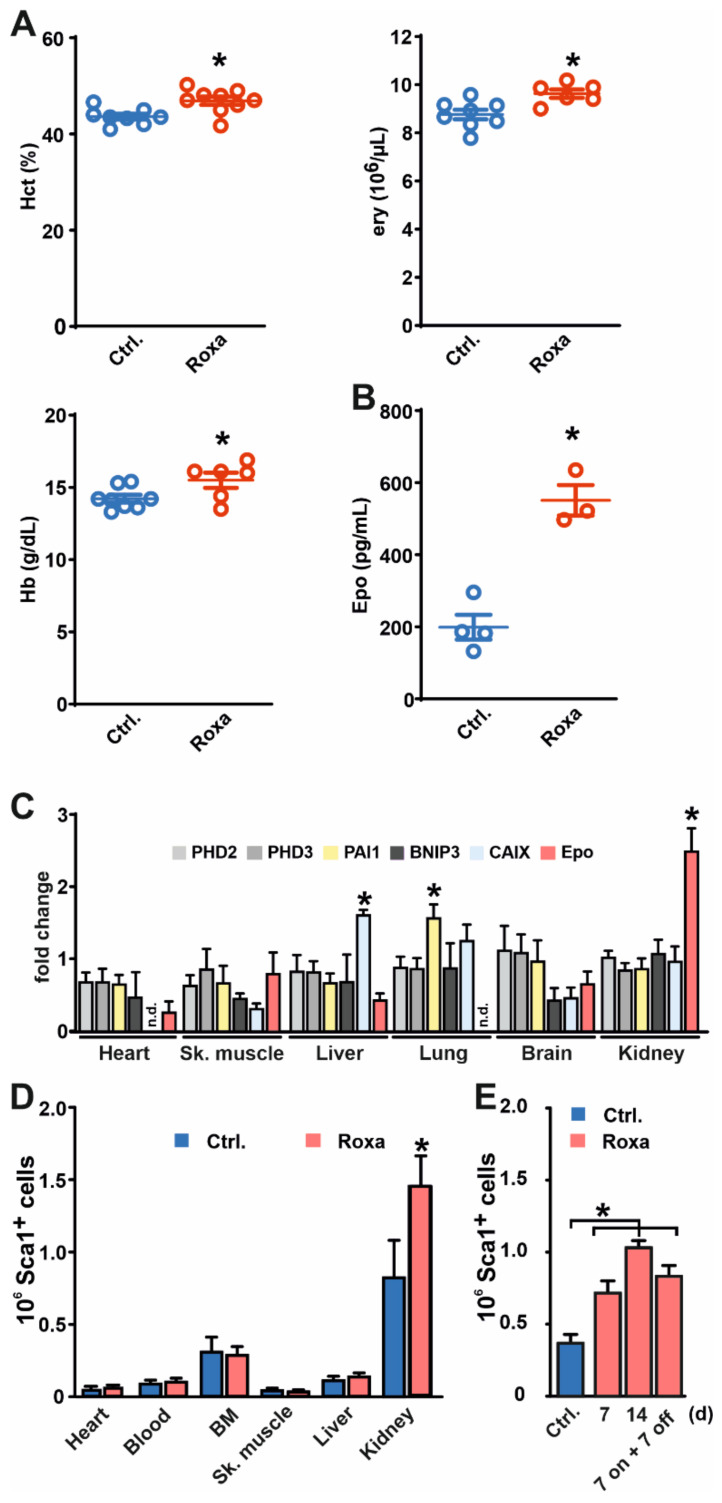
Increased number of Sca-1^+^ cells in the kidneys following treatment with roxadustat. Mice were treated with roxadustat or solvent as a control (Ctrl.) for seven days. Subsequently, (**A**) hematocrit (Hct), erythrocyte (ery), and hemoglobin (Hb) concentration as well as (**B**) erythropoietin (Epo) plasma levels were determined. (**C**) Organ mRNA levels are shown as a fold change over the control-treated animals (*n* = 4–8 mice). (**D**) Organs/tissues were harvested from solvent control and roxadustat-treated mice (*n* = 9 in each group) and Sca-1^+^ cells were quantified. (**E**) Number of Sca-1+ cells isolated from the kidneys of mice treated with solvent (Ctrl.) or with roxadustat, as indicated (*n* = 6 mice per group). Mean values ± SEM are shown. * *p* < 0.05.

We treated mice with roxadustat or a solvent control via i.p. injections for seven days. Roxadustat was dosed to significantly increase the hematocrit, hemoglobin, and erythrocyte (Figure 1A). These changes in red blood cell parameters were accompanied by increased plasma Epo levels (Figure 1B). Quantification of known hypoxia-inducible genes (*PHD2*, *PHD3*, *PAI1*, *BNIP3*, *CAIX*, and *Epo*) in several organs, i.e., the heart, skeletal muscle, liver, lungs, brain, and kidneys, demonstrated a kidney tropism of the roxadustat response (Figure 1C). In the kidneys, Epo was the only significantly regulated gene. Aside from the increase in Epo in the kidneys, we found increased mRNA levels of the carbonic anhydrase IX (CAIX) and plasminogen activator inhibitor 1 (PAI1) in the liver and lungs, respectively, but not in the kidneys. All other analyzed transcripts, i.e., PHD2, PHD3, and BNIP3, remained unchanged in roxadustat-treated mice. This selective induction of HIF target genes, and especially Epo, by roxadustat is in line with the purpose of developing this drug to increase hematocrit.

To assess the effects of roxadustat on MSC-like cells, we employed FACS of dissociated whole organs and specific tissues to define the overall frequency of Sca-1^+^ cells in seven days roxadustat versus solvent control-treated mice. In all organs/tissues analyzed, we found Sca-1^+^ cells in untreated animals. However, exclusively in the kidneys, the number of Sca-1^+^ cells increased significantly after roxadustat treatment (Figure 1D). Increasing the duration of roxadustat treatment from seven days to 14 days slightly further increased the number of Sca-1^+^ cells (Figure 1E). In contrast, treating mice with roxadustat for seven days and leaving the animals untreated for another seven days did not significantly change the number of Sca-1^+^ cells compared to the seven-day-treated animals, indicating that roxadustat-induced cells remain in the kidneys.

**Figure 2:**
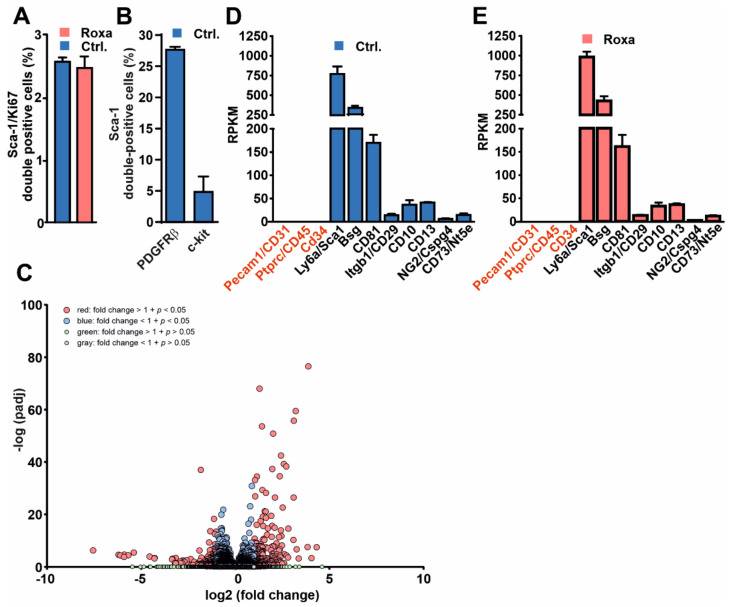
Characterization of kidney-derived Sca-1^+^ cells. Co-expression of (**A**) Sca-1/Ki 67 (*n* = 3 mice per group) and (**B**) Sca-1/c-kit as well as Sca-1/PDGFRβ (*n* = 6 mice) in Sca-1^+^ cells isolated from the kidneys of solvent- (Ctrl.) or roxadustat (seven days)-treated mice. (**C**) Volcano plot of 179 significantly up- and 97 significantly downregulated mRNAs in Sca-1^+^ cells isolated from mice treated with roxadustat for seven days compared to solvent-treated mice. (**D**,**E**) Selected nonmesenchymal (red) and mesenchymal (black) stem cell markers in Sca-1^+^ cells isolated from solvent or roxadustat-treated mice; reads per kilobase million (RPKM). Mean values ± SEM are shown.

Interestingly, Bapst et al., recently described an increase in stem cell markers, including Sca-1, in conditionally immortalized REP cells upon incubating the cells to permissive conditions that inhibit large-T expression [14]. Overlapping Sca-1 and Epo mRNA expression in interstitial REP cells was also confirmed in vivo using an EPO reporter mouse line. To further characterize the Sca-1^+^ cell population identified in our study, we next isolated these cells and analyzed proliferation and various surface markers as well as their transcriptome. Ki-67 protein has been widely used as a proliferation marker. To obtain insight into the proliferation capacity of the Sca-1^+^ cells, we isolated Sca-1^+^ cells, fixed the cells, and stained them for Ki-67. Sca-1^+^ cells showed low levels of the Ki67-proliferation marker when isolated from both solvent and roxadustat-treated mice (Figure 2A). To further characterize the Sca-1^+^ cell population, we next quantified their expression of c-kit and PDGFRβ by FACS analysis. Only a small number of the Sca-1^+^ cells co-expressed c-kit, a marker that is expressed on hematopoietic stem cells. In contrast, Sca-1^+^ cells co-expressed PDGFRβ, which is commonly found in REP cells (Figure 2B), demonstrating that the isolated Sca-1^+^ cells contain a mix of different Sca-1^+^ subpopulations. Transcriptome analysis of the Sca-1^+^ cells revealed 179 significantly up- and 97 significantly downregulated RNAs in cells isolated from roxadustat-treated mice compared to solvent-treated mice (Figure 2C and Table S1). A reactome-based pathway analysis revealed that Sca-1^+^ cells isolated from roxadustat-treated mice exhibit a significant upregulation of pathways regulating extracellular matrix organization and collagen biosynthesis compared to the controls (Table S2). Analyzing mesenchymal cell markers in the RNAseq data, we found expression of typical mesenchymal stem cell markers, e.g., Sca-1, Bsg, CD81, CD29, CD10, CD13, NG2, and CD73, which, however, did not differ between the cells isolated from roxadustat-treated mice and solvent-treated mice. Sca-1^+^ cells did not express CD31, CD45, and CD34 (Figure 2D,E), which are markers for endothelial cells, white blood cells, and multipotent progenitor cells, respectively.

### 3.2. Sca-1^+^ Cells Display a Mesenchymal Stem-Cell-like Phenotype

**Figure 3:**
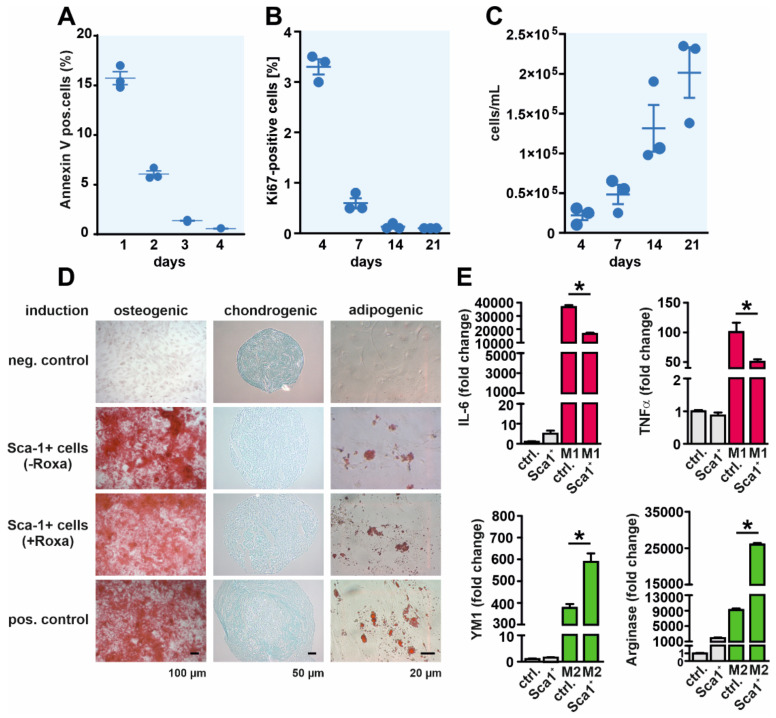
Sca-1^+^ cells display a mesenchymal stem-cell-like phenotype. (**A**) Annexin V and (**B**) Ki67 levels, and (**C**) cell counts after isolation of Sca-1^+^ cells at the time points indicated. (**D**) Trilineage (osteogenic, chondrogenic, and adipogenic) differentiation of Sca-1^+^ kidney-derived cells. Bone marrow-derived mesenchymal stem cells were used as a control. Sca-1^+^ cells cultured in a conventional cell culture medium without stimulation served as a negative control. (**E**) RNA levels of M1 (IL-6 and TNFα) and M2 (YM1 and arginase) markers (*n* = 3 biological replicates) of bone marrow-derived macrophages (BMDM), which were nonpolarized (gray bars), M1- polarized (red bars), or M2-polarized (green bars) in the presence of the control or a conditioned cell culture medium obtained from L929 or Sca-1^+^ cells, respectively. Mean values ± SEM are shown; * *p* < 0.05.

To further characterize the identified cell population, isolated Sca-1^+^ cells were cultivated for up to three weeks in vitro. The cells were plated on conventional cell culture dishes and nonadherent cells were washed away. To obtain insight into cell viability and cell proliferation, respectively, Annexin V and Ki-67 levels were analyzed by FACS staining. Annexin V-positive cells declined from 15% down to roughly 1% within four days after isolation (Figure 3A). Sca-1^+^ cells proliferated for up to 21 days in culture, but with a significant decrease in Ki67-positive cells over time (Figure 3B,C), indicating a low proliferation capacity of the cells under the applied cell culture conditions.

MSCs are defined by their capacity to differentiate into at least bone, cartilage, and fat [19]. To further analyze their stem cell properties, we cultivated and differentiated Sca-1^+^ cells in the presence or absence of roxadustat (Figure 3D). In parallel, we differentiated bone marrow-derived MSCs, which served as a positive control for the differentiation protocol. Bone marrow-derived MSCs fulfilled the criterium of the characteristic trilineage differentiation potential. Sca-1^+^ cells differentiated efficiently into the osteogenic and adipogenic lineage compared to nondifferentiated cells independent of the presence of roxadustat. Chondrogenic differentiation, however, was less efficient compared to the bone marrow-derived MSCs. Aside from their differentiation potential, MSCs have the ability to alter innate immune function, including macrophage polarization, via the secretion of immunomodulatory factors [20]. Therefore, we incubated murine bone marrow-derived-macrophages during M1 and M2 polarization with a conditioned cell culture medium obtained from Sca-1^+^ cells. As a control, we additionally treated macrophages with conditioned medium from L929 cells to exclude Sca-1^+^ cell-independent effects derived from the conditioned medium (Figure 3E). Afterwards, M1 (IL-6 and TNFα) and M2 (Ym-1 and arginase) polarization markers were analyzed by RT-qPCR. Both markers increased upon stimulation with LPS and IFN-γ (M1-polarization) or IL-4 (M2-polarization), respectively. Treatment with a Sca-1^+^-conditioned medium significantly decreased the M1 polarization, whereas M2 polarization was stimulated. Taken together, we found typical characteristics of MSC-like cells in the Sca-1^+^ cell population, i.e., plastic adherence, MSC-like marker expression, trilineage differentiation potential, and macrophage M2 polarization ability.

### 3.3. Kidney-Derived Sca-1^+^ Cells Produce Epo

**Figure 4:**
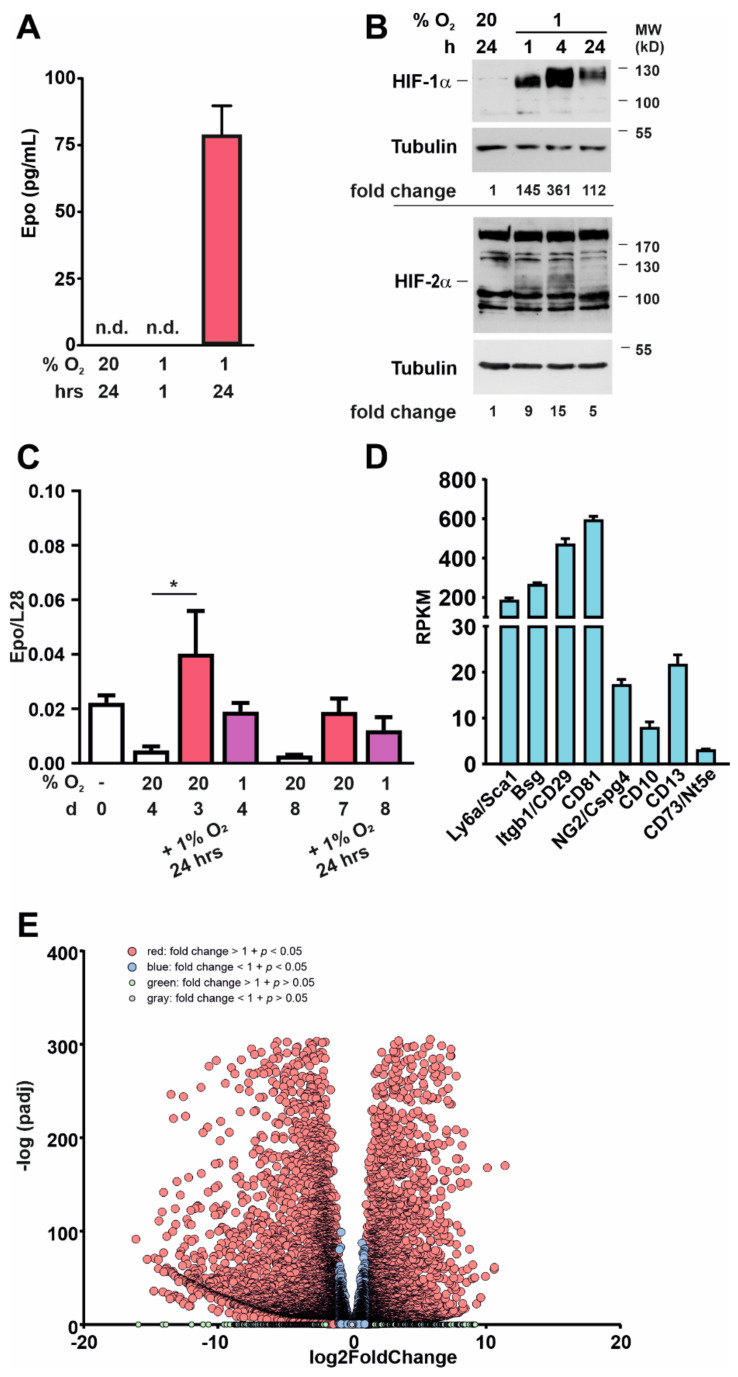
Kidney-derived Sca-1^+^ cells produce Epo. (**A**) Epo protein levels in supernatants obtained from Sca-1^+^ cells cultured in normoxia or hypoxia (*n* = 3 biological replicates). (**B**) HIF-1α and HIF-2α protein levels in Sca-1^+^ kidney-derived cells after exposure to normoxia (20% O_2_) or hypoxia (1% O_2_). Numbers below the blots indicate the fold change of the ratio of HIF-1α/Tubulin and HIF-2α/Tubulin in 1% O_2_ to the respective 20% O_2_ control. (**C**) Epo RNA levels in Sca-1^+^ cells that were incubated either at 20% O_2_ of 1% O_2_ as indicated (*n* = 4 biological replicates). (**D**) Mesenchymal stem cell markers in renal Sca-1^+^ cells on day 21 of culture; reads per kilobase million (RPKM). (**E**) Volcano plot of 4592 significantly up- and 5376 significantly downregulated mRNAs in kidney-derived Sca-1^+^ cells on day 21 in culture compared to day 0. Mean values ± SEM are shown. * *p* < 0.05.

To gain insight into their Epo-producing capacity, we analyzed Sca-1^+^ cells on day 4 after isolation and determined Epo protein levels in the cell culture supernatants after incubating the cells in normoxia or hypoxia (Figure 4A). Epo levels were nondetectable in normoxia, whereas in hypoxia Epo increased in the supernatants. Epo expression was paralleled by stabilization of HIF-1α and HIF-2α protein in hypoxia (Figure 4B). The ability to produce Epo decreased over time. On day 8 after isolation, Epo was no longer detectable in the supernatants of the cells, whether incubated in normoxia or hypoxia (data not shown). Significantly increased RNA levels of Epo were detectable in Sca-1^+^ cells on day 4 but not day 8 after incubation in 1% O_2_ for 24 h (Figure 4C). Although there was a trend that Epo RNA levels were preserved when keeping the cells at 1% O_2_ compared to 20% O_2_ after isolation, this was not statistically significant.

Changes over time were seen in gene expression, as analyzed by RNAseq performed with Sca-1^+^ cells on day 0 and day 21 after isolation. Although the mesenchymal stem cell markers Sca-1, Bsg, CD29, CD81, NG2, CD10, and CD13 were still expressed (Figure 4D), RNAseq revealed 4592 significantly upregulated and 5376 significantly downregulated RNAs on day 21 compared to day 0 (Figure 4E and Table S3). This included upregulation of RNAs associated with a fibroblastic phenotype, including TGFβ, TGFβR1, TGFβR2, Acta2, and FN1. A reactome-based pathway analysis of the regulated genes indicated a significant stimulation of pathways associated with intracellular cytoskeleton and extracellular matrix reorganization (Table S4). The conversion of Epo-producing cells into myofibroblast-like cells and a coincident loss of Epo-producing ability are commonly found in vivo and in vitro and are believed to be a major cause of renal anemia and fibrosis in CKD patients [21,22].

## 4. Discussion

PHIs have been developed and clinically approved for treating patients with renal anemia [7]. Injecting mice with the PHI roxadustat resulted in a significant increase in hematocrit, erythrocyte count, and hemoglobin concentration, which was accompanied by elevated Epo plasma levels. Aside from increased Epo mRNA levels in the kidneys, the response in other organs was minor. This organ tropism of roxadustat is within the scope of an anemia-related drug, which should mainly affect the physiological production sites of endogenous Epo, i.e., the liver and kidneys [23,24]. Roxadustat potently inhibits all three PHD isoforms [25], but is selective for PHDs over factor-inhibiting HIF (FIH), an asparaginyl hydroxylase capable of regulating HIF transcriptional activity. Therefore, the relative specificity of roxadustat towards PHDs results in incomplete mimicry of the hypoxic response. For example, PHD3, which is a robust HIF target gene in hypoxia, responds much less to PHD-specific inhibition [6]. In line, no induction of PHD3 mRNA was observed in the roxadustat-treated animals, whereas other HIF-specific targets like Epo, PAI1, and CAIX were induced in the kidneys, lungs, and liver, respectively.

During embryogenesis, a MSC population is detectable in the kidneys [26]. These cells are, in part, responsible for the development of the renal interstitium [27,28]. Moreover, MSC-like cell populations were detected in the murine adult kidneys [29]. Most interestingly, cells isolated by a similar approach to that applied in our study were found to express Sca-1 and were able to differentiate into adipogenic and osteogenic lineages [30,31] as well as nestin^+^ neuronal cells [32] upon stimulation in vitro. Among those were the so-called MSCs-E4 cells, which are able to produce Epo in vitro after exposure to hypoxia [30]. However, the Epo response of the MSCs-E4 cells was mild, with protein levels in the cell culture supernatants barely detectable by ELISA. Compared to our study, Plotkin et al., used a cell isolation protocol, which selected for adherent cells after tissue digestion. In contrast, we enriched Sca-1^+^ mesenchymal cells by using a MACS sorting protocol. The resulting cell population robustly produced Epo even though the capacity of Epo production was limited to the first days after isolation. Sca-1 expression was also described in a conditionally immortalized cell line, which relied on a recently established transgenic REP cell fate mapping mouse model [12,14]. In contrast to the cell population described here, these cells did not show a trilineage differentiation potential. Instead, the cells were capable of neurogenic differentiation.

Sca-1 is a member of the Ly6 family that is expressed on the surface of hematopoietic and mesenchymal stem cells in mice [33]. It is a glycosyl phosphatidylinositol-anchored protein containing cysteine rich domains that interact through disulfide bridges to create the distinctive “three-fingered” structural domain found in all family members. Sca-1 was long believed to be a marker for a cardiac progenitor cell population that is able to differentiate into fully functional cardiomyocytes. Fate mapping by the use of different genetic lineage-tracing mouse models recently revealed that cardiac Sca-1^+^ cells represent just a subset of endothelial cells that are able to expand in response to pathological stress [34]. The Sca-1^+^ cardiac progenitor cells are thus not intrinsic stem cells for myocardial renewal and repair [35]. Sca-1 knockout mice have been described in the past [36,37]. These animals show a surprising lack of phenotype, with no indications for anemia. Hematopoiesis is essentially normal aside from a minor lineage skewing, affecting mostly megakaryocytes, and resulting in mild thrombocytopenia [38].

With the present study, we cannot answer the question of whether Sca-1 is functionally involved in the development of Epo-producing cells. Despite its wide expression pattern, the mechanism by which Sca-1 acts still remains unclear. Keeping in mind the mild phenotype of Sca-1 knockout mice, Sca-1 expression in the Epo cell population likely reflects a distinct state in the continuum of mesenchymal cell plasticity rather than a functional mechanism. The origin of the Sca-1^+^ Epo-producing cell population remains elusive. Essentially, all Sca-1^+^ cells described so far are mesoderm derivatives, reflecting the nature of the Sca-1^+^ interstitial cells. This is in contrast to other Epo-producing cell types that occur during embryogenesis and are thought to derive from the neural crest [39]. Plasticity of the Sca-1^+^ mesenchymal cells would include de- or transdifferentiation processes. Conditional immortalization of a recently generated REP cell line indicated that a kidney-derived mesenchymal cell population is indeed capable of regressing to a Sca-1^+^-expressing cell population [14]. Considering the neural-crest-derived Epo-producing cells described in the literature, our data support the concept that the Epo-producing cell pool as a whole might be composed of various lineages. There is evidence that the Epo cell population is not a fixed entity but rather fluctuates and shows marked heterogeneity. Depending on the induction of Epo production by, for example, hypoxemia, inhibition of HIFα degradation, etc., the pool of cells recruited seems to vary regarding the subpopulation and localization [40]. Gene expression analysis likewise revealed tremendous changes within days of culturing the Sca-1^+^ cells. This coincides with a small time window in which the Sca-1^+^ cells were able to produce Epo after isolation, in line with the rare examples for the successful establishment of REP cell lines, in which cells retain the ability to produce low levels of Epo in vitro over time [12]. In vitro expansion thus results in a population that is not representative of the cell input, which might be due to the fact that the cell-specific in vivo conditions are difficult to mimic in vitro. In vivo, Epo production ability is thought to be inactivated by myofibroblastic transdifferentiation. Therefore, renal anemia and fibrosis are tightly related to each other during chronic kidney disease progression [22,41]. Using an immortalized cell line derived from renal Epo-producing cells, Sato et al., demonstrate that cell-autonomous TGFβ signaling and epigenetic silencing are involved in myofibroblastic differentiation [21]. In line with this, the transcriptomic profile of our cells changes dramatically up to day 21 after isolation, with changes in genes involved in the cytoskeleton and extracellular matrix architecture.

The identified Sca-1^+^ cells add to our understanding of the plasticity of renal Epo-producing cells, an area that has thus far been difficult to investigate. Likewise, our study augments the knowledge about the powerful mechanisms of the PHI roxadustat to correct anemia in CKD patients. Aside from mimicking hypoxia-inducible gene expression, roxadustat seems to be able to recruit Sca-1^+^ cells to the Epo-producing cell pool in the kidneys, eventually supporting erythropoiesis.

## Data Availability

The RNA seq data presented in this study are openly available in the Gene Expression Omnibus repository (GEO data http://www.ncbi.nlm.nih.gov/geo/query/acc.cgi?acc%20=%20GSE150669GSE150669, accessed on 17 February 2022).

## Supplementary Materials

The following supporting information can be downloaded at: https://www.mdpi.com/article/10.3390/cells11040753/s1: Table S1: List of significantly (*p* < 0.05) regulated genes in Sca-1^+^ cells isolated from the kidneys of roxadustat-treated mice (7 days, 33 mg/kg body weight, i.p.) compared to Sca-1^+^ cells isolated from solvent-treated control mice (7 days, vehicle, i.p.). Listed are the 50 most up-regulated and the 50 most downregulated genes, as determined by RNAseq; Table S2: List of significantly (*p* < 0.05) enriched pathways in Sca-1^+^ MACS-sorted cells isolated from the kidneys of control mice (seven days, vehicle injection) in comparison to roxadustat-injected mice (7 days, 33 mg/kg body weight). Table S3: List of significantly (*p* < 0.05) regulated genes in Sca-1^+^ MACS-sorted cells after 21 days in culture compared to Sca-1^+^ MACS-sorted cells on the day of isolation. Listed are the 100 most upregulated and the 100 most downregulated genes, as determined by RNAseq; Table S4: List of significantly (*p* < 0.05) enriched pathways in Sca-1^+^ MACS-sorted cells isolated from the kidneys directly after isolation in comparison to Sca-1^+^ MACS-sorted cells on day 21 after isolation in cell culture.
