# Supplementary material for: The HIFα-Stabilizing Drug Roxadustat Increases the Number of Renal Epo-Producing Sca-1+ Cells
Source: Cells. 2022 Feb 21;11(4):753. doi: 10.3390/cells11040753 (PMC8869801; doi:10.3390/cells11040753)
Supplement: Supplementary file 1 [file cells-11-00753-s001.zip › cells-1581441-supplementary.pdf]

Supplemetary Table S1: List of significantly ($p < 0.05$) regulated genes in Sca-1$^+$ cells isolated from the kidneys of roxadustat-treated mice (7 days, 33 mg/kg body weight, i.p.) compared to Sca-1$^+$ cells isolated from solvent-treated control mice (7 days, vehicle, i.p.). Listed are the 50 most induced and the 50 most down-regulated genes as determined by RNAseq.

Gene Name	Gene	log2 fold change
chemokine (C-C motif) ligand 8	Ccl8	4,32
predicted gene 15564	Gm15564	4,05
periostin, osteoblast specific factor	Postn	3,88
sialic acid binding Ig-like lectin 1, sialoadhesin	Siglec1	3,84
triggering receptor expressed on myeloid cells 2	Trem2	3,37
chemokine (C-X-C motif) ligand 5	Cxcl5	3,26
RIKEN cDNA 9030619P08 gene	9030619P08Rik	3,21
collagen, type III, alpha 1	Col3a1	3,11
tissue inhibitor of metalloproteinase 1	Timp1	3,10
fibrillin 2	Fbn2	3,09
leucine rich repeat containing 17	Lrrc17	2,82
hedgehog interacting protein-like 1	Hhipl1	2,80
serine (or cysteine) peptidase inhibitor, clade B, member 2	Serpinb2	2,73
chemokine (C-C motif) ligand 6	Ccl6	2,71
H19, imprinted maternally expressed transcript	H19	2,61
mitochondria localized glutamic acid rich protein	Mgarp	2,59
collagen, type I, alpha 2	Col1a2	2,58
lumican	Lum	2,52
collagen, type I, alpha 1	Col1a1	2,48
lymphatic vessel endothelial hyaluronan receptor 1	Lyve1	2,45
lysyl oxidase	Lox	2,43
cartilage intermediate layer protein, nucleotide pyrophosphohydrolase	Cilp	2,42
leucyl-tRNA synthetase, mitochondrial	Lars2	2,39
membrane-spanning 4-domains, subfamily A, member 7	Ms4a7	2,39
arginase, liver	Arg1	2,38
RNA imprinted and accumulated in nucleus	Rian	2,37
carbonic anhydrase 9	Car9	2,36
protease, serine 35	Prss35	2,34
WAP four-disulfide core domain 17	Wfdc17	2,32
a disintegrin and metallopeptidase domain 12 (meltrin alpha)	Adam12	2,32
predicted gene, 43921	Gm43921	2,31
matrix metallopeptidase 3	Mmp3	2,31
coagulation factor XIII, A1 subunit	F13a1	2,19
alpha-2-macroglobulin	A2m	2,19
epiregulin	Ereg	2,19
olfactory receptor 1033	Olfr1033	2,17
ficolin A	Fcna	2,16
membrane-spanning 4-domains, subfamily A, member 6D	Ms4a6d	2,14
reticulocalbin 3, EF-hand calcium binding domain	Rcn3	2,11
microfibrillar-associated protein 4	Mfap4	2,10
predicted gene, 16754	Gm16754	2,10
carboxypeptidase X 1 (M14 family)	Cpxm1	2,09
miRNA containing gene	Mirg	2,08
tenascin C	Tnc	2,07
arylsulfatase i	Arsi	2,03
transmembrane protein 45a	Tmem45a	2,02
lysozyme 2	Lyz2	2,01
asporin	Aspn	2,00
sushi-repeat-containing protein, X-linked 2	Srpx2	1,98
collagen, type V, alpha 3	Col5a3	1,98

Gene Name	Gene	log2 fold change
D site albumin promoter binding protein	Dbp	-1,29
UDP-GlcNAc:betaGal beta-1,3-N-acetylglucosaminyltransferase 3	B3gnt3	-1,29
latent transforming growth factor beta binding protein 4	Ltbp4	-1,31
archaelysin family metallopeptidase 1	Amz1	-1,31
aquaporin 2	Aqp2	-1,33
endoglin	Eng	-1,35
phosphatidylinositol-4-phosphate 3-kinase catalytic subunit type 2 beta	Pik3c2b	-1,35
cilia and flagella associated protein 54	Cfap54	-1,35
aquaporin 4	Aqp4	-1,37
glycerophosphodiester phosphodiesterase domain containing 3	Gdpd3	-1,37
histocompatibility 2, Q region locus 6	H2-Q6	-1,38
proprotein convertase subtilisin/kexin type 9	Pcsk9	-1,39
arylsulfatase J	Arsj	-1,39
lymphocyte protein tyrosine kinase	Lck	-1,43
heat shock protein 12B	Hspa12b	-1,47
cytochrome P450, family 2, subfamily a, polypeptide 5	Cyp2a5	-1,51
leiomodin 1 (smooth muscle)	Lmod1	-1,51
serine (or cysteine) peptidase inhibitor, clade A, member 1D	Serpina1d	-1,53
solute carrier family 14 (urea transporter), member 2	Slc14a2	-1,56
C-type lectin domain family 18, member A	Clec18a	-1,59
chemokine (C-X-C motif) receptor 6	Cxcr6	-1,59
carbonic anhydrase 3	Car3	-1,78
predicted gene 4759	Gm4759	-1,80
heme oxygenase 1	Hmox1	-1,83
AF4/FMR2 family, member 3	Aff3	-1,88
basic helix-loop-helix family, member e41	Bhlhe41	-1,90
expressed sequence AA465934	AA465934	-2,18
cytochrome P450, family 4, subfamily a, polypeptide 12a	Cyp4a12a	-2,37
formiminotransferase cyclodeaminase	Ftcd	-2,59
SH3 domain containing ring finger 3	Sh3rf3	-2,69
polycystic kidney disease 1 like 1	Pkd1l1	-2,78
predicted gene 11868	Gm11868	-2,79
predicted gene 4353	Gm4353	-2,82
NEL-like 2	Nell2	-2,91
glutathione S-transferase, alpha 1 (Ya)	Gsta1	-2,93
tubulin, beta 4A class IVA	Tubb4a	-3,15
valosin containing protein, related sequence	Vcp-rs	-3,16
predicted gene 7292	Gm7292	-3,21
predicted gene 15920	Gm15920	-3,34
proliferating cell nuclear antigen pseudogene 2	Pcna-ps2	-3,36
predicted gene 11942	Gm11942	-4,28
predicted pseudogene 6916	RP23-412J12.1	-4,29
predicted pseudogene 10320	Gm10320	-4,55
UDP glycosyltransferase 1 family, polypeptide A10	Ugt1a10	-5,39
predicted gene 10263	Gm10263	-5,68
UDP glucuronosyltransferase 1 family, polypeptide A8	Ugt1a8	-5,89
ribosomal protein S2, pseudogene 13	Rps2-ps13	-5,91
cDNA sequence BC018473	BC018473	-6,11
UDP glucuronosyltransferase 1 family, polypeptide A9	Ugt1a9	-6,20
predicted gene 15375	Gm15375	-7,55

Supplementary Table S2: List of significantly ($p < 0.05$) enriched pathways in Sca-1$^+$ MACS-sorted cells isolated from the kidneys of roxadustat-injected mice (seven days, 33 mg/kg body weight) in comparison to control-treated mice (seven days, solvent control). The input list contained all genes that were at least log2 1.5-fold induced or down-regulated as determined by RNAseq.

**pathway up-regulated**	**p-value**
Assembly of collagen fibrils and other multimeric structures	1.09E-3
MET activates PTK2 signaling	1.95E-3
ECM proteoglycans	2.3E-3
Integrin cell surface interactions	2.76E-3
Degradation of the extracellular matrix	3.16E-3
Extracellular matrix organization	3.22E-3
Collagen degradation	4.12E-3
Non-integrin membrane-ECM interactions	5.24E-3
Collagen chain trimerization	5.24E-3
MET promotes cell motility	6.69E-3
Collagen formation	7.8E-3
Crosslinking of collagen fibrils	8.83E-3
Platelet Adhesion to exposed collagen	1.49E-2
GP1b-IX-V activation signalling	1.76E-2
Anchoring fibril formation	2.99E-2
Collagen biosynthesis and modifying enzymes	3.51E-2
Elastic fibre formation	3.83E-2

**pathway down-regulated**	**p-value**
Passive transport by Aquaporins	1.66E-2
Insertion of tail-anchored proteins into the endoplasmic reticulum membrane	2.39E-2
Glucuronidation	3.85E-2

Supplementary Table S3: List of significantly ($p < 0.05$) regulated genes in to Sca-1⁺ MACS-sorted cells after 21 days in culture compared to Sca-1⁺ MACS-sorted cells at the day of isolation. Listed are the 100 most induced and the 100 most down-regulated genes as determined by RNAseq.

Gene Name	Gene	log2 fold change
collagen, type XI, alpha 1	Col11a1	11,40
cyclin dependent kinase inhibitor 2A	Cdkn2a	10,62
actin, gamma 2, smooth muscle, enteric	Actg2	10,60
latent transforming growth factor beta binding protein 2	Ltbp2	10,09
G protein-coupled receptor 176	Gpr176	9,95
solute carrier family 1 (high affinity aspartate/glutamate transporter), member 6	Slc1a6	9,60
solute carrier family 5 (choline transporter), member 7	Slc5a7	9,58
carboxypeptidase A6	Cpa6	9,29
AHNAK nucleoprotein 2	Ahnak2	9,18
cellular communication network factor 5	Wisp2	9,15
growth differentiation factor 6	Gdf6	9,14
cellular communication network factor 3	Nov	9,13
lysyl oxidase	Lox	9,06
collagen, type XII, alpha 1	Col12a1	9,04
predicted gene, 43921	Gm43921	9,01
serine (or cysteine) peptidase inhibitor, clade B, member 9b	Serpinb9b	8,97
periostin, osteoblast specific factor	Postn	8,92
polypeptide N-acetylgalactosaminyltransferase 17	Wbscr17	8,76
fibulin 2	Fbln2	8,73
collagen, type I, alpha 1	Col1a1	8,61
sema domain, immunoglobulin domain (Ig), short basic domain, secreted, (semaphorin) 3A	Sema3a	8,61
epiregulin	Ereg	8,54
adenylate cyclase activating polypeptide 1 receptor 1	Adcyap1r1	8,44
forkhead box G1	Foxg1	8,44
BMP-binding endothelial regulator	Bmper	8,43
fibronectin 1	Fn1	8,35
golgi associated kinase 1B	Fam198b	8,33
claudin 11	Cldn11	8,29
chloride channel accessory 3A1	Clca3a1	8,27
collagen, type VIII, alpha 1	Col8a1	8,26
zinc finger protein 469	Gm22	8,25
biglycan	Bgn	8,24
bone morphogenetic protein/retinoic acid inducible neural specific 3	Brinp3	8,23
a disintegrin and metallopeptidase domain 23	Adam23	8,20
transmembrane protein 45a	Tmem45a	8,12
procollagen-proline, 2-oxoglutarate 4-dioxygenase (proline 4-hydroxylase), alpha polypeptide III	P4ha3	8,11
protocadherin 9	Pcdh9	8,11
glycoprotein (transmembrane) nmb	Gpnmb	8,07
cytochrome P450, family 1, subfamily b, polypeptide 1	Cyp1b1	8,05
RIKEN cDNA 3110039M20 gene	3110039M20Rik	8,02
CD80 antigen	Cd80	8,01
mannose receptor, C type 2	Mrc2	8,00
collagen, type I, alpha 2	Col1a2	7,97
tenascin C	Tnc	7,96
WAP four-disulfide core domain 18	Wfdc18	7,96
cadherin 11	Cdh11	7,94
lysyl oxidase-like 2	Loxl2	7,93
small proline-rich protein 3	Sprr3	7,86
matrix metallopeptidase 2	Mmp2	7,86
vestigial like family member 3	Vgll3	7,85
angiopoietin 4	Angpt4	7,83

thrombospondin 2	Thbs2	7,81
platelet-derived growth factor receptor-like	Pdgfrl	7,80
paired related homeobox 1	Prrx1	7,80
fibrillin 2	Fbn2	7,79
bone morphogenic protein/retinoic acid inducible neural specific 1	Brinp1	7,78
a disintegrin and metallopeptidase domain 12	Adam12	7,77
FK506 binding protein 10	Fkbp10	7,76
tissue inhibitor of metalloproteinase 1	Timp1	7,76
Na+/K+ transporting ATPase interacting 3	Nkain3	7,73
inhibin beta-A	Inhba	7,69
CD5 antigen-like	Cd5l	7,69
protein tyrosine phosphatase, receptor type, V	Ptprv	7,64
retinoic acid early transcript delta	Raet1d	7,58
tropomyosin 2, beta	Tpm2	7,58
forkhead box S1	Foxs1	7,58
oxidized low density lipoprotein (lectin-like) receptor 1	Olr1	7,53
macrophage receptor with collagenous structure	Marco	7,52
predicted gene, 37644	Gm37644	7,50
ventral anterior homeobox 2	Vax2	7,49
predicted gene 43517	3110039M20Rik	7,49
predicted gene, 32647	RP23-231J2.1	7,48
predicted gene 6806	Gm6806	7,47
high mobility group AT-hook 2	Hmga2	7,46
UDP-Gal:betaGlcNAc beta 1,3-galactosyltransferase, polypeptide 1	B3galt1	7,43
low density lipoprotein receptor-related protein 1	Lrp1	7,42
pleiotrophin	Ptn	7,40
short stature homeobox 2	Shox2	7,39
a disintegrin and metallopeptidase domain 19 (meltrin beta)	Adam19	7,38
cathepsin K	Ctsk	7,38
matrix Gla protein	Mgp	7,36
protocadherin 10	Pcdh10	7,34
fibroblast growth factor 2	Fgf2	7,32
killer cell lectin-like receptor, subfamily A, member 4	Klra4	7,32
matrix metallopeptidase 14 (membrane-inserted)	Mmp14	7,31
actin, alpha 2, smooth muscle, aorta	Acta2	7,29
Na+/K+ transporting ATPase interacting 4	Nkain4	7,29
S100 calcium binding protein A6 (calcyclin)	S100a6	7,28
microtubule-associated protein 6	Map6	7,26
scavenger receptor cysteine rich family, 5 domains	Ssc5d	7,26
collagen, type V, alpha 1	Col5a1	7,25
integrin alpha 11	Itga11	7,25
cytotoxic T lymphocyte-associated protein 2 alpha	Ctla2a	7,25
flavin containing monooxygenase 6	Fmo6	7,24
syntrophin, gamma 1	Sntg1	7,24
EYA transcriptional coactivator and phosphatase 4	Eya4	7,22
layilin	Layn	7,20
cadherin 18	Cdh18	7,18
lumican	Lum	7,17
desmin	Des	7,17

Gene Name	Gene	log2 fold change
oxoglutarate (alpha-ketoglutarate) receptor 1	Oxgr1	-11,73
solute carrier family 22 (organic anion transporter), member 7	Slc22a7	-11,73
solute carrier family 44, member 4	Slc44a4	-11,74
transmembrane protease, serine 2	Tmprss2	-11,75
UDP galactosyltransferase 8A	Ugt8a	-11,77
barttin CLCNK type accessory beta subunit	Bsnd	-11,77
epithelial cell adhesion molecule	Epcam	-11,79

dimethylglycine dehydrogenase precursor	Dmgdh	-11,80
retinol dehydrogenase 16	Rdh16	-11,82
PZP, alpha-2-macroglobulin like	Pzp	-11,84
solute carrier family 5 (sodium/glucose cotransporter), member1	Slc5a1	-11,89
claudin 8	Cldn8	-11,91
cadherin 1	Cdh1	-11,95
N-acetyltransferase 8 (GCN5-related)	Nat8	-11,95
gamma-glutamyltransferase 1	Ggt1	-11,96
cytochrome P450, family 4, subfamily a, polypeptide 10	Cyp4a10	-11,97
sodium channel, nonvoltage-gated 1 gamma	Scnn1g	-11,98
serine (or cysteine) peptidase inhibitor, clade F, member 2	Serpinf2	-11,98
sclerostin domain containing 1	Sostdc1	-11,99
aldehyde dehydrogenase 8 family, member A1	Aldh8a1	-12,01
polymeric immunoglobulin receptor	Pigr	-12,01
arylacetamide deacetylase	Aadac	-12,03
solute carrier family 7 (cationic amino acid transporter, y+ system), member 9	Slc7a9	-12,03
C-type lectin domain family 2, member h	Clec2h	-12,05
glucose-6-phosphatase, catalytic	G6pc	-12,06
protein C	Proc	-12,06
coagulation factor XIII, beta subunit	F13b	-12,07
solute carrier family 6 (neurotransmitter transporter, GABA), member 13	Slc6a13	-12,12
guanylate cyclase activator 2b (retina)	Guca2b	-12,13
solute carrier family 22, member 28	Slc22a28	-12,13
meprin 1 beta	Mep1b	-12,14
acyl-CoA synthetase medium-chain family member 5	Acsm5	-12,14
phenylalanine hydroxylase	Pah	-12,16
sulfotransferase family 1D, member 1	Sult1d1	-12,18
hydroxy-delta-5-steroid dehydrogenase, 3 beta- and steroid delta-isomerase 2	Hsd3b2	-12,19
myelin and lymphocyte protein, T cell differentiation protein	Mal	-12,20
transmembrane protein 72	Tmem72	-12,21
betaine-homocysteine methyltransferase 2	Bhmt2	-12,21
solute carrier family 6 (neurotransmitter transporter), member 19	Slc6a19	-12,24
solute carrier family 12, member 3	Slc12a3	-12,29
cytochrome P450, family 2, subfamily j, polypeptide 13	Cyp2j13	-12,31
carbonic anhydrase 4	Car4	-12,34
chloride channel, voltage-sensitive Kb	Clcnkb	-12,40
solute carrier family 12, member 1	Slc12a1	-12,41
carboxylesterase 2C	Ces2c	-12,44
solute carrier family 22 (organic cation transporter), member 22	Slc22a22	-12,46
PDZ domain containing 1	Pdzk1	-12,48
hydroxyacid oxidase 2	Hao2	-12,48
solute carrier family 26, member 4	Slc26a4	-12,49
integrin beta 6	Itgb6	-12,50
secreted phosphoprotein 2	Spp2	-12,54
fructose bisphosphatase 1	Fbp1	-12,56
ATP-binding cassette, sub-family A (ABC1), member 13	Abca13	-12,57
pipecolic acid oxidase	Pipox	-12,63
Rhesus blood group-associated C glycoprotein	Rhcg	-12,68
cytochrome P450, family 2, subfamily j, polypeptide 11	Cyp2j11	-12,72
carboxylesterase 1D	Ces1d	-12,76
predicted gene, 19950	RP23-306P12.2	-12,77
UDP glycosyltransferases 3 family, polypeptide A1	Ugt3a1	-12,78
solute carrier family 47, member 1	Slc47a1	-12,80
solute carrier family 22 (organic cation transporter), member 1	Slc22a1	-12,81
serum/glucocorticoid regulated kinase 2	Sgk2	-12,83
defensin beta 1	Defb1	-12,83
UDP glycosyltransferases 3 family, polypeptide A2	Ugt3a2	-12,85
solute carrier family 13 (sodium/sulfate symporters), member 1	Slc13a1	-12,86

cytochrome P450, family 2, subfamily a, polypeptide 4	Cyp2a4	-12,90
WAP four-disulfide core domain 15B	Wfdc15b	-12,94
solute carrier family 7, (cationic amino acid transporter, y+ system) member 13	Slc7a13	-12,95
klotho	Kl	-13,05
cytochrome P450, family 2, subfamily e, polypeptide 1	Cyp2e1	-13,08
solute carrier family 17 (sodium phosphate), member 1	Slc17a1	-13,14
solute carrier family 22 (organic anion transporter), member 8	Slc22a8	-13,16
indolethylamine N-methyltransferase	Inmt	-13,18
calbindin 1	Calb1	-13,18
calcium-sensing receptor	Casr	-13,19
cytochrome P450, family 2, subfamily j, polypeptide 5	Cyp2j5	-13,32
solute carrier family 22 (organic cation transporter), member 2	Slc22a2	-13,34
D-amino acid oxidase	Dao	-13,37
solute carrier family 13 (sodium-dependent dicarboxylate transporter), member 3	Slc13a3	-13,37
solute carrier family 22 (organic anion transporter), member 6	Slc22a6	-13,42
transmembrane protein 174	Tmem174	-13,45
kidney expressed gene 1	Keg1	-13,49
kallikrein 1	Klk1	-13,57
meprin 1 alpha	Mep1a	-13,67
solute carrier organic anion transporter family, member 1a1	Slco1a1	-13,78
napsin A aspartic peptidase	Napsa	-13,81
UDP glucuronosyltransferase 2 family, polypeptide B38	Ugt2b38	-13,82
cytochrome P450, family 4, subfamily b, polypeptide 1	Cyp4b1	-13,87
serine peptidase inhibitor, Kazal type 1	Spink1	-13,89
aldolase B, fructose-bisphosphate	Aldob	-14,04
FXYD domain-containing ion transport regulator 2	Fxyd2	-14,05
hepatic nuclear factor 4, alpha	Hnf4a	-14,05
solute carrier family 34 (sodium phosphate), member 1	Slc34a1	-14,10
aldo-keto reductase family 1, member C21	Akr1c21	-14,16
collectrin, amino acid transport regulator	Tmem27	-14,40
solute carrier family 17 (sodium phosphate), member 3	Slc17a3	-14,43
myo-inositol oxygenase	Miox	-14,43
carboxylesterase 1F	Ces1f	-14,72
acyl-CoA synthetase medium-chain family member 2	Acsm2	-14,77
solute carrier family 27 (fatty acid transporter), member 2	Slc27a2	-15,27
phosphoenolpyruvate carboxykinase 1, cytosolic	Pck1	-16,11

Supplementary Table S4: List of significantly ($p < 0.05$) enriched pathways in Sca-1$^+$ MACS-sorted cells isolated from the kidney directly after isolation in comparison to Sca-1$^+$ MACS-sorted cells on day 21 after isolation in cell culture. The input list contained all genes that were at least log2 1.5-fold induced or down-regulated as determined by RNAseq.

**pathway up-regulated**	**p-value**
Collagen degradation	3.88E-4
Assembly of collagen fibrils and other multimeric structures	2.97E-3
Non-integrin membrane-ECM interactions	3.43E-3
Collagen chain trimerization	3.43E-3
MET activates PTK2 signaling	4.94E-3
Extracellular matrix organization	5.04E-3
Degradation of the extracellular matrix	5.24E-3
Crosslinking of collagen fibrils	1.19E-2
ECM proteoglycans	1.24E-2
Invadopodia formation	1.35E-2
Elastic fibre formation	1.99E-2
MET promotes cell motility	1.99E-2
Integrin cell surface interactions	2.62E-2
Collagen formation	2.81E-2
Collagen biosynthesis and modifying enzymes	4.43E-2

**pathway down-regulated**	**p-value**
Abacavir transmembrane transport	1.25E-2
